# Emotional Blunting in Depression in the PREDDICT Clinical Trial: Inflammation-Stratified Augmentation of Vortioxetine With Celecoxib

**DOI:** 10.1093/ijnp/pyad066

**Published:** 2024-03-05

**Authors:** Emma Sampson, Erhan Kavakbasi, Natalie T Mills, Hikaru Hori, K Oliver Schubert, Célia Fourrier, Bernhard T Baune

**Affiliations:** Discipline of Psychiatry, Adelaide Medical School, University of Adelaide, Adelaide, Australia; Department of Psychiatry, University Hospital Münster, University of Münster, Münster, Germany; Discipline of Psychiatry, Adelaide Medical School, University of Adelaide, Adelaide, Australia; Department of Psychiatry, Faculty of Medicine, Fukuoka University, Fukuoka City, Japan; Discipline of Psychiatry, Adelaide Medical School, University of Adelaide, Adelaide, Australia; Northern Adelaide Mental Health Service, Salisbury, Australia; Headspace Adelaide Early Psychosis, Sonder, Adelaide, Australia; Discipline of Psychiatry, Adelaide Medical School, University of Adelaide, Adelaide, Australia; Discipline of Psychiatry, Adelaide Medical School, University of Adelaide, Adelaide, Australia; Department of Psychiatry, University Hospital Münster, University of Münster, Münster, Germany; Department of Psychiatry, Melbourne Medical School, The University of Melbourne, Melbourne, Australia; The Florey Institute of Neuroscience and Mental Health, The University of Melbourne, Parkville, VIC, Australia

**Keywords:** Major depressive disorder, emotional blunting, randomized controlled trial, anti-inflammatory treatment, celecoxib

## Abstract

**Background:**

Emotional symptoms are recognized as a key feature in individuals with major depressive disorder. Previously, emotional blunting has been described both as a side effect of antidepressant treatment and as a symptom of depression. Little is known about the change of emotional blunting during antidepressant treatment.

**Methods:**

The PREDDICT trial is a randomized, placebo-controlled, 6-week trial on the augmentation of vortioxetine with the anti-inflammatory agent celecoxib or placebo. Presently we report on exploratory secondary outcomes of changes in emotional blunting in depression assessed with the Oxford Depression Questionnaire (ODQ) total score and subscores from baseline to 8-week, 3-month, and 6-month follow-up assessments.

**Results:**

In the whole group, there was a significant improvement in the ODQ total score and all subscores after 8 weeks. After stratification of participants into the treatment groups, the ODQ total score as well as subscores related to emotional blunting as a symptom of depression (reduction in positive emotions, not caring) improved between baseline and all follow-up time points in both treatment groups. Changes in subscores considered as a side effect of antidepressants (general reduction in emotions, emotional detachment) were inconclusive in both treatment groups. Overall, the placebo-augmented group showed slightly better results in changes of emotional blunting scores than the celecoxib group as did those with elevated inflammation at screening, regardless of treatment group.

**Conclusions:**

This analysis suggests favorable effects of vortioxetine on emotional blunting in both short- and long-term course. The beneficial impact of vortioxetine on emotional blunting was weaker in celecoxib-augmented patients compared with placebo, possibly due to pharmacokinetic interactions.

**Clinical Trials Registration:** Australian New Zealand Clinical Trials Registry (ANZCTR), ACTRN12617000527369p. Registered on 11 April 2017, http://www.anzctr.org.au/Trial/Registration/TrialReview.aspx?ACTRN=12617000527369p.

**Significance Statement**

Although emotional symptoms are the most recognizable feature of depression, research into symptoms of emotional blunting and how they respond to antidepressant treatment is limited. To our knowledge, no studies have yet examined emotional blunting response to the antidepressant vortioxetine beyond 8 weeks of treatment, nor has the response of these symptoms to anti-inflammatory treatment been tested. Our study addresses this gap and reports benefits of vortioxetine treatment up to 35 weeks from baseline on these symptoms but also reveals no further advantage with the addition of 6 weeks of high-dose anti-inflammatory celecoxib medication. In fact, the synergistic effects of combined treatment may lead to worse outcomes in this context than participants treated with vortioxetine alone.

## Introduction

Major depressive disorder (MDD) is one of the greatest causes of burden of disease affecting more than 300 million people worldwide). Individuals suffering from depression experience impairment of both physical and mental health and functioning (Liu et al., 2020). Besides depressed mood, lack of drive, and reduction of interests, emotional dysfunction is one of the core symptoms of depression (Morris et al., 2009). Disturbed emotional experience in depression is also associated with cognitive dysfunction in terms of concentration and memory deficits as well as rumination (Dehn and Beblo, 2019). Individuals with depression show a negative bias in precepting emotional expressions, which is also correlated to the extent of rumination (Raes et al., 2006). Behavioral and imaging studies revealed attenuated response to happy faces as well as increased brain activation to sad faces in depression patients in emotion-related regions. This disturbed processing of emotional information has also been shown after symptom remission and in heathy controls at higher risk for depression (Leppänen, 2006). Abnormalities and difficulties in emotion regulation have been shown to be predictive for severity of depression symptoms (Atherton et al., 2015; Crisan and Nechita, 2022). There is also some evidence that reduction in positive emotionality may predict poor outcome in MDD (Morris et al., 2009). Clinically, emotional symptoms of depression manifest as frequent or persistent depressed, dreadful, worried, or sad mood (American Psychiatric Association, 2013). Additionally, significantly reduced ability to feel pleasure or enjoyment for usual interests and hobbies and, in more pronounced form, inability to enjoy or feel anything positive or negative or complete emotional unresponsiveness has been recognized as a core symptomatic feature in depressed individuals (Kendler, 2016).

The Montgomery-Åsberg Depression Rating Scale (MADRS) as one of the most frequently used depression rating scales includes 3 items that primarily assess mood and emotions: reported and apparent sadness, as well as inability to feel positive or negative emotions (Montgomery and Åsberg, 1979). Emotional blunting has been described as emotional numbness with an inability to feel negative or positive emotions, reduced responsiveness to emotional stimuli, as well as feelings of detachment from others. Although these phenomena are common in depressed patients, emotional blunting may also be related to antidepressant use as a side effect of antidepressant agents, which has led to some controversy (Christensen et al., 2022). In earlier concepts, reduced emotional responsiveness was described as a side effect in patients treated with selective serotonin reuptake inhibitors (SSRIs), where emotional blunting was present in patients suffering from sexual dysfunction induced by SSRIs. In this study with a relatively small sample size (n = 15), 80% of patients reported blunting of a wide range of emotions, which have been assumed to be treatment related (Opbroek et al., 2002). In support of these earlier findings, a more recent report shows that emotional blunting has been listed as one of the common reasons for discontinuation of antidepressants in patient surveys (Rosenblat et al., 2019). However, because emotional blunting scores highly correlated with depression severity, it has been concluded that emotional blunting cannot solely be attributed to a medication side effect but should also be considered as a residual symptom of depression (Goodwin et al., 2017). The latter has also been discussed in another study, where emotional blunting scores correlated with MADRS scores and were found to be lower in remitted than in nonremitted patients (Aşçibaşi et al., 2020). Emotional blunting remains an important concern during antidepressant treatment (Jawad et al., 2023).

The Oxford Depression Questionnaire (ODQ) has been validated for measurement of emotional blunting in patients receiving vortioxetine (Christensen et al., 2021). A large survey including 669 patients under treatment and 150 recovered patients revealed presence of emotional blunting in about 46% of patients on antidepressant medication (Goodwin et al., 2017). In this study the highest prevalence of emotional blunting has been found in SSRI (varying 43%–47% depending on agent, citalopram, paroxetine, escitalopram, fluoxetine) and serotonin norepinephrine reuptake inhibitors venlafaxine, desvenlafaxine, and duloxetine treated participants (Goodwin et al., 2017). Emotional blunting has been present in all examined agents, including mirtazapine and amitriptyline, and the lowest percentage was found in bupropion (33%) treated individuals (Goodwin et al., 2017). The authors did not include vortioxetine in this survey. Previously, vortioxetine has yielded significant improvement of anhedonia in patients with MDD (Cao et al., 2019; McIntyre et al., 2021). Improvement in anhedonia has also resulted in improved functioning (McIntyre et al., 2021). Additionally, to our knowledge, vortioxetine is the only antidepressant agent that has been demonstrated to improve emotional blunting in patients with MDD. In an uncontrolled, single-arm study, patients with MDD and incomplete response to SSRI or serotonin norepinephrine reuptake inhibitors were switched to 8 weeks of vortioxetine 10–20 mg daily. Vortioxetine led to significant improvement in ODQ total score and all subscores (Fagiolini et al., 2021). To date there are no published data on the impact of vortioxetine on emotional blunting in the long-term course beyond 8 weeks. Neither have there been studies investigating the impact of antidepressant augmentation with anti-inflammatory agents on emotional blunting. In the present analysis we report on exploratory results from the randomized, placebo controlled, double-blind PREDDICT trial on the efficacy of anti-inflammatory 6-week augmentation of vortioxetine with celecoxib vs vortioxetine plus placebo. The study protocol and the primary results were previously published in detail (Fourrier et al., 2018; Baune et al., 2021). This manuscript focuses on the changes of emotional blunting symptoms over an 8-month total period measured by the ODQ total score and subscores as secondary outcome parameters. The present analysis focuses on the following objectives:

- To investigate change in emotional blunting total score and subscores after 8 weeks of vortioxetine treatment
- To analyze the differences of changes in emotional blunting between celecoxib- and placebo-augmented patients
- To study the long-term changes in emotional blunting over 3 and 6 months following the 6-week augmentation with celecoxib and placebo

## METHODS

### Study Design

**Figure 1.**
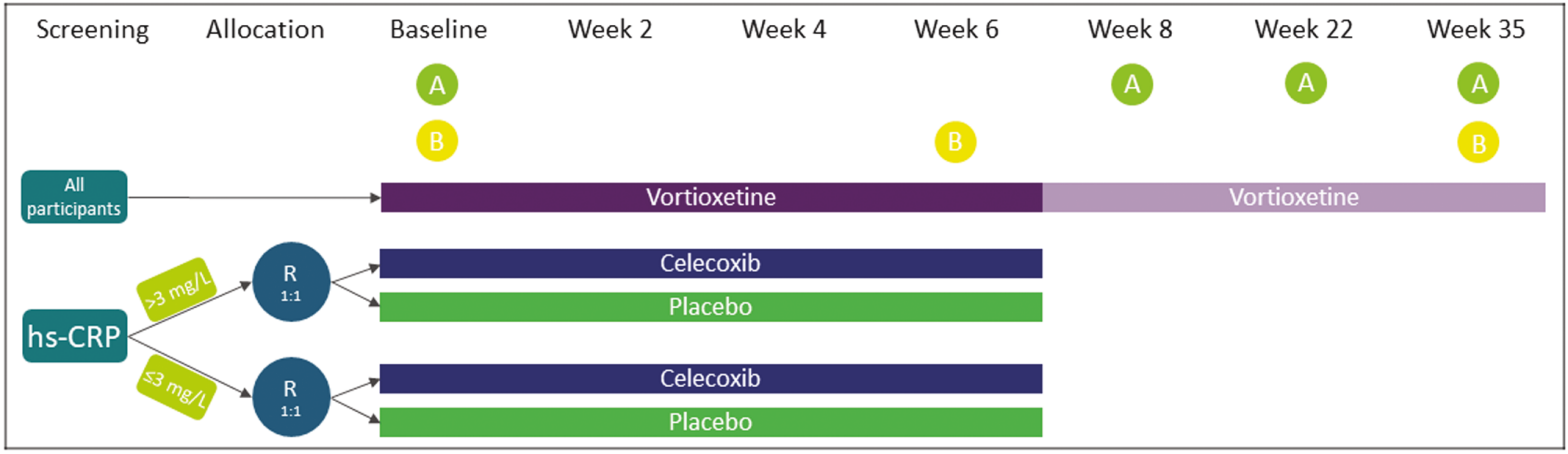
PREDDICT RCT schedule of treatments and assessments^*a*^. ^*a*^*Adapted from**Kavakbasi* et al.*, 2023*. A, administration of the Oxford Depression Questionnaire (ODQ) assessment; B, collection of peripheral venous blood; R, randomization. Participation in the trial and continued treatment with vortioxetine was optional from week 8. Week 22 and week 35 indicate 3-month follow-up from week 8 and 6-month follow-up from week 8, respectively. Other assessments at all listed time points were performed but are not listed here because they are outside the scope of this publication. For full list and timings of all assessments, see (Fourrier et al., 2018).

Emotional blunting data were collected as a secondary outcome of the PREDDICT study, a double-blinded, randomized, controlled trial (RCT) with superiority framework. The trial protocol was previously published, including full inclusion and exclusion criteria (Fourrier et al., 2018), as have the reports of the main outcome and selected secondary outcomes (Baune et al., 2021; Kavakbasi et al., 2023; Sampson et al., 2023). Briefly, participants with current MDD validated with the Mini-international Neuropsychiatric Interview (v5.0 for DSM-IV; Sheehan et al., 1998), stratified by pretreatment inflammatory marker high-sensitivity C-reactive protein (hsCRP), were randomized to receive vortioxetine augmented with either 400 mg of the anti-inflammatory celecoxib or placebo for a duration of 6 weeks, as shown in Figure 1. Dosages of vortioxetine in the celecoxib-augmented group were reduced by half during the 6-week RCT period relative to the placebo-augmented group according to the manufacturer’s guidelines (Trintellix product monograph, page 1 of 44, Lundbeck) due to known interactions between celecoxib and vortioxetine; specifically, celecoxib is a strong inhibitor of the CYP2D6 enzyme, which metabolizes vortioxetine from its bioactive form to inactive waste metabolites (Werner et al., 2003; Chen et al., 2013; Areberg et al., 2014; Fourrier et al., 2018). Dosage adjustments did not lead to differences in efficacy as measured by overall MDD severity or different side effect profiles between the 2 groups (Baune et al., 2021). Following the RCT phase, vortioxetine was provided for up to an additional 29 weeks, with assessments completed at 8 weeks post baseline and then again at 22 weeks from baseline (i.e., 3-month follow-up from week 8) and 35 weeks from baseline (i.e., 6-month follow-up from week 8).

The study was conducted at the University of Adelaide, Australia, between December 2017 and April 2020 and was approved by the Human Research Ethics Committees of the Royal Adelaide Hospital and the University of Adelaide (reference no. R20170320 HREC/17/RAH/111) and registered on the Australian New Zealand Clinical Trials Registry, ACTRN12617000527369 (www.anzctr.org.au/Trial/Registration/TrialReview.aspx?ACTRN=12617000527369p).

### Outcome

The ODQ is a self-administered questionnaire used to assess symptoms related to emotional blunting in patients with MDD (Price et al., 2012; Goodwin et al., 2017). It is rated on a 5-point Likert scale, with higher scores indicating greater impairment. As well as a total score ranging from 20 to 100, the questionnaire scores 4 core dimensions, each of which have scoring ranges of 5 to 25: general reduction in emotions (GR), reduction in positive emotions (RP), emotional detachment from others (ED), and not caring (NC). An additional dimension of antidepressant as cause (AC), scored from 6 to 30, is calculated but does not contribute to the ODQ total score. The AC section was not completed at baseline.

Additionally, 2 subtotals are calculated, combining RP and NC for an RP-NC score and combining GR and ED for a GR-ED score, as recommended by the authors of the original development and validation study, as their analysis suggested that there may be a distinction wherein RP-NC scores may correlate with emotional blunting as a symptom of depression, whereas GR-ED may correlate with emotional blunting as a side effect of antidepressant medication (Price et al., 2012).

### Statistical Methods

Linear mixed effects models for repeated measures with random intercept and participant as random effect were used to assess change over time for the aforementioned variables using the package lmerTest, which extends the lme4 package, in R (version 4.2.1, R Foundation for Statistical Computing (Bates et al., 2015; Kuznetsova et al., 2017). Three versions of each model were constructed; the first did not contain treatment group in the model (presented in Table 2), whereas the second included treatment group as an interaction term (presented in Tables 3 and 4 below), and the third included treatment group and hsCRP strata as interaction terms. All models included age and sex as covariates. Overall time-by-treatment group effects were tested on the constructed models with lmerTest’s ANOVA function, which executes Type III ANOVA with Satterthwaite’s method (Kuznetsova et al., 2017).

**Table 1.** Baseline Characteristics of the Study Population With Data Available for Analysis

Variable		All	Vortioxetine + placebo	Vortioxetine + celecoxib	Group difference [F(df), p] or [OR (95%CI), p]
Total participants, PREDDICT		N = 119	n = 60	n = 59	
Total participants included in present analyses		N = 88	n = 45	n = 43	
Age	Median (IQR)	47 (33, 55)	47 (29, 54)	49 (39.5, 57)	0.703 (1,86), 0.404
Sex	Male n (%)	29 (33)	14 (31)	15 (35)	0.845 (0.315, 2.254), 0.844
	Female n (%)	59 (67)	31 (69)	28 (65)	
Treatment-resistant depression	Yes n (%)	71 (81)	36 (80)	35 (81)	1.093 (0.331, 3.666), 1.000
ODQ total score	Mean (SD)	66.68 (15.03)	66.40 (16.39)	66.98 (13.66)	0.032 (1,86), 0.858
ODQ GR	Median (IQR)	17 (13, 21.25)	17 (13, 22)	17 (13.5, 21)	0.013 (1,86), 0.909
ODQ RP	Median (IQR)	21 (19, 24)	22 (19, 24)	21 (19, 24.5)	0.090 (1,86), 0.765
ODQ ED	Median (IQR)	12 (7.75, 15.25)	12 (7, 16)	11 (8, 14.5)	0.127 (1,86), 0.723
ODQ NC	Median (IQR)	17 (14, 20)	18 (14, 20)	17 (15, 20)	0.316 (1,86), 0.576
ODQ GR-ED	Mean (SD)	29 (8.724)	29.13 (9.360)	28.86 (8.114)	0.021 (1,86), 0.884
ODQ RP-NC	Median (IQR)	39 (34, 44)	39 (33, 44)	39 (35.5, 43)	0.227 (1,86), 0.635
MADRS score	Median (IQR)	27.5 (24, 32)	26 (21, 30)	29 (26, 34.5)	9.908 (1,86), 0.002

Abbreviations: CI = confidence interval; ED = emotional detachment from others; GR = general reduction in emotions; IQR = interquartile range; MADRS = Montgomery–Åsberg Depression Rating Scale; NC = not caring; ODQ = Oxford Depression Questionnaire; OR = odds ratio; RP = reduction in positive emotions. Data are expressed in n (%), mean (SD) for normally distributed data, and median (IQR) for data non-normally distributed in at least 1 measured group at baseline. Group differences calculated with linear models for continuous variables and Fisher exact tests for binomial variables. Treatment resistant depression recorded if participant had 2 or more failed trials of MDD treatment that were of adequate dosage and duration.

**Table 2.** Change in ODQ Measures Between Baseline and Week 8, Whole Cohort

Measure	Estimate (95% CI)	*P* value
ODQ total score	−9.363 (−13.245 to −5.481)	<.001
ODQ GR	−1.413 (−2.728 to −0.099)	.035
ODQ RP	−3.029 (−4.314 to −1.744)	<.001
ODQ ED	−1.547 (−2.613 to −0.481)	.005
ODQ NC	−3.375 (−4.569 to −2.182)	<.001
ODQ GR-ED	−2.955 (−4.941 to −0.970)	.004
ODQ RP-NC	−6.397 (−8.665 to −4.130)	<.001

Abbreviations: CI = confidence interval; ED = emotional detachment from others; GR = general reduction in emotions; NC = not caring; ODQ = Oxford Depression Questionnaire; RP = reduction in positive emotions. Estimates and *P* values refer to the difference between baseline and week 8. Covariates include participant age and sex.

**Table 3.** Change in ODQ Measures Between Baseline and Week 8 by Treatment Group

Measure	Treatment group	Estimate (95% CI)	*P* value
ODQ total score	Vortioxetine + placebo	−11.739 (−17.062 to −6.416)	<.001
	Vortioxetine + celecoxib	−6.570 (−12.273 to −0.866)	.024
	Group difference (vortioxetine and celecoxib—vortioxetine and placebo)	5.169 (−2.630 to 12.969)	.193
ODQ GR	Vortioxetine + placebo	−1.392 (−3.203 to 0.418)	.131
	Vortioxetine + celecoxib	−1.419 (−3.356 to 0.518)	.150
	Group difference (vortioxetine and celecoxib—vortioxetine and placebo)	−0.026 (−2.677 to 2.624)	.984
ODQ RP	Vortioxetine + placebo	−3.755 (−5.519 to −1.991)	<.001
	Vortioxetine + celecoxib	−2.187 (−4.077 to −0.298)	.024
	Group difference (vortioxetine and celecoxib—vortioxetine and placebo)	1.568 (−1.017 to 4.153)	.233
ODQ ED	Vortioxetine + placebo	−2.059 (−3.507 to −0.610)	.006
	Vortioxetine + celecoxib	−0.992 (−2.544 to 0.561)	.209
	Group difference (vortioxetine and celecoxib—vortioxetine and placebo)	1.067 (−1.056 to 3.190)	.322
ODQ NC	Vortioxetine + placebo	−4.520 (−6.136 to −2.903)	<.001
	Vortioxetine + celecoxib	−1.992 (−3.723 to −0.261)	.024
	Group difference (vortioxetine and celecoxib—vortioxetine and placebo)	2.528 (0.159 to 4.896)	.037
ODQ GR-ED	Vortioxetine + placebo	−3.459 (−6.182 to −0.736)	.013
	Vortioxetine + celecoxib	−2.388 (−5.305 to 0.529)	.108
	Group difference (vortioxetine and celecoxib—vortioxetine and placebo)	1.071 (−2.919 to 5.060)	.597
ODQ RP-NC	Vortioxetine + placebo	−8.274 (−11.372 to −5.176)	<.001
	Vortioxetine + celecoxib	−4.168 (−7.488 to −0.848)	.014
	Group difference (vortioxetine and celecoxib—vortioxetine and placebo)	4.106 (−0.434 to 8.646)	.076

Abbreviations: CI = confidence interval; ED = emotional detachment from others; GR = general reduction in emotions; NC = not caring; ODQ = Oxford Depression Questionnaire; RP = reduction in positive emotions. Estimates and *P* values refer to the difference between baseline and week 8 for each treatment group, and the difference between the baseline to week 8 change in the vortioxetine plus celecoxib vs the baseline to week 8 change in the vortioxetine plus placebo group for the “group difference” test. Covariates include participant age and sex.

**Table 4.** Change in ODQ Measures Between Baseline And 3-Month Follow-Up and Baseline and 6-Month Follow-Up, by Treatment Group^*a*^

Measure			Estimate (95% CI)	*P* value
ODQ total score	Overall time-by-treatment interaction		F(df) = 0.739 (3,189.842)^	.530^
	Vortioxetine + placebo	Baseline to 3-month follow-up	−9.121 (−14.691 to −3.552)	.002
		Baseline to 6-month follow-up	−12.142 (−18.086 to −6.198)	<.001
	Vortioxetine + celecoxib	Baseline to 3-month follow-up	−8.851 (−15.063 to −2.640)	.005
		Baseline to 6-month follow-up	−8.887 (−15.535 to −2.239)	.009
ODQ GR	Overall time-by-treatment interaction		F(df) = 0.277 (3,193.833)^	.842^
	Vortioxetine + placebo	Baseline to 3-month follow-up	−1.680 (−3.570 to 0.210)	.081
		Baseline to 6-month follow-up	−1.783 (−3.799 to 0.233)	.083
	Vortioxetine + celecoxib	Baseline to 3-month follow-up	−2.840 (−4.939 to −0.740)	.008
		Baseline to 6-month follow-up	−1.971 (−4.213 to 0.272)	.085
ODQ RP	Overall time-by-treatment interaction		F(df) = 0.536 (3,191.046)^	.658^
	Vortioxetine + placebo	Baseline to 3-month follow-up	−3.488 (−5.333 to −1.643)	<.001
		Baseline to 6-month follow-up	−3.775 (−5.745 to −1.806)	<.001
	vortioxetine + celecoxib	Baseline to 3-month follow-up	−2.341 (−4.398 to −0.284)	.026
		Baseline to 6-month follow-up	−3.225 (−5.427 to −1.024)	.004
ODQ ED	Overall time-by-treatment interaction		F(df) = 1.659 (3,187.621)^	.177^
	Vortioxetine + placebo	Baseline to 3-month follow-up	−1.521 (−3.037 to −0.004)	.049
		Baseline to 6-month follow-up	−2.887 (−4.506 to −1.268)	<.001
	Vortioxetine + celecoxib	Baseline to 3-month follow-up	−0.611 (−2.304 to 1.082)	.478
		Baseline to 6-month follow-up	−0.170 (−1.983 to 1.643)	.854
ODQ NC	Overall time-by-treatment interaction		F(df) = 2.399 (3,189.562)^	.070^
	Vortioxetine + placebo	Baseline to 3-month follow-up	−2.517 (−4.207 to −0.827)	.004
		Baseline to 6-month follow-up	−3.795 (−5.598 to −1.992)	<.001
	Vortioxetine + celecoxib	Baseline to 3-month follow-up	−3.212 (−5.093 to −1.331)	<.001
		Baseline to 6-month follow-up	−3.699 (−5.711 to −1.687)	<.001
ODQ GR-ED	Overall time-by-treatment interaction		F(df) = 0.597 (3,190.525)^	.618^
	Vortioxetine + placebo	Baseline to 3-month follow-up	−3.161 (−6.009 to −0.313)	.030
		Baseline to 6-month follow-up	−4.617 (−7.656 to −1.578)	.003
	Vortioxetine + celecoxib	Baseline to 3-month follow-up	−3.410 (−6.582 to −0.237)	.035
		Baseline to 6-month follow-up	−2.091 (−5.485 to 1.302)	.226
ODQ RP-NC	Overall time-by-treatment interaction		F(df) = 1.228 (3,189.404)^	.301^
	Vortioxetine + placebo	Baseline to 3-month follow-up	−5.973 (−9.215 to −2.730)	<.001
		Baseline to 6-month follow-up	−7.532 (−10.992 to −4.072)	<.001
	vortioxetine + celecoxib	Baseline to 3-month follow-up	−5.466 (−9.082 to −1.850)	.003
		Baseline to 6-month follow-up	−6.832 (−10.703 to −2.962)	<.001
ODQ antidepressant as cause	Overall time-by-treatment interaction		F(df) = 0.480 (2,100.888)	.620^
	Vortioxetine + placebo	Week 8 to 3-month follow-up	1.0462 (−0.556 to 2.648)	.198
		Week 8 to 6-month follow-up	−0.822 (−2.553 to 0.910)	.349
	Vortioxetine + celecoxib	Week 8 to 3-month follow-up	0.3819 (−1.507 to 2.271)	.689
		Week 8 to 6-month follow-up	−0.241 (−2.271 to 1.789)	.815

Abbreviations: CI = confidence interval; ED = emotional detachment from others; GR = general reduction in emotions; NC = not caring; ODQ = Oxford Depression Questionnaire; RP = reduction in positive emotions.

^*a*^Week 8 is the timepoint used as first comparison for the antidepressant as cause measure. ^F, df, and *P* values indicated with “^” refer to time-by-treatment group interaction calculated with type III ANOVA with Satterthwaite’s method. “Time” variable includes baseline, week 8, 3-month follow-up, and 6-month follow-up observations, except for the “antidepressant as cause measure,” which includes week 8, 3-month follow-up and 6-month follow-up observations only. Estimates and *P* values refer to the difference in means. Covariates include participant age and sex.

## Results

Summaries of sociodemographic characteristics and outcome measures are given in Table 1. ODQ data were available for 88 participants at baseline, 72 participants at week 8, 67 participants at 3-month follow-up, and 55 participants at 6-month follow-up. At baseline, no significant group differences between the vortioxetine plus placebo vs vortioxetine plus celecoxib-treated strata in any of the ODQ measures were observed; however, the baseline MADRS score was higher in the celecoxib-augmented group than in the placebo-augmented group (Table 1).

**Figure 2.**
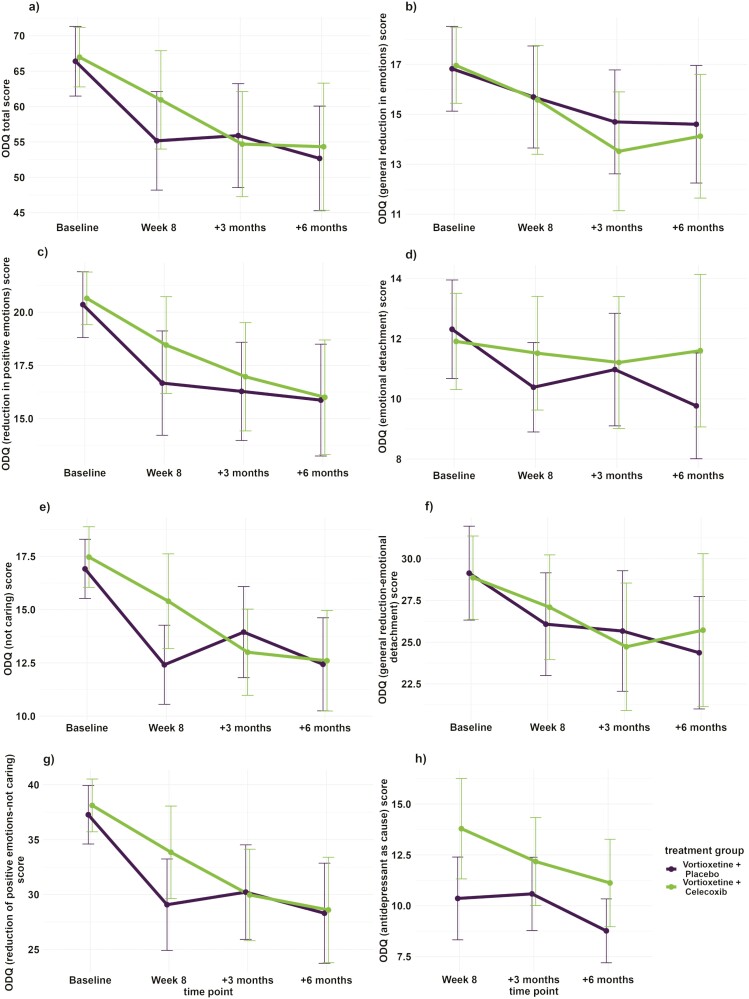
Change over time in Oxford Depression Questionnaire (ODQ) score and subscales over time by treatment group. Plots of the mean of vortioxetine + placebo and vortioxetine + celecoxib scores at baseline, week 8, 3-month follow-up, and 6-month follow-up time points for *a* ODQ total score, *b* ODQ general reduction in emotions score, *c* ODQ reduction in positive emotions score, *d* ODQ emotional detachment score, *e* ODQ not caring score, *f* ODQ GR-ED score, *g* ODQ RP-NC score, and *h* ODQ antidepressant as cause score. Error bars = 95% confidence interval (CI).

Firstly, we investigated the change in ODQ scores from baseline to week 8 in all vortioxetine-treated participants in the entire cohort. This revealed that the ODQ total score and all subscales demonstrated a significant decrease over this period (Table 2). When the participants were stratified into placebo and celecoxib-augmented treatment groups, the vortioxetine plus placebo–treated group maintained a significant reduction between baseline and week 8 for the ODQ total score, for 3 out of the 4 core dimension scores, and on both subtotal scores, with only the GR dimension failing to achieve a significant change over time in this period. On the other hand, the vortioxetine plus celecoxib–treated group demonstrated a significant reduction over time for the ODQ total score, RP, NC, and RP-NC subscales related to apathy and anhedonia but no significant change over the same period for the ODQ GR, ED, and GR-ED subscales related to social interest and general emotionality (Table 3; Figure 2).

Secondly, when analyzing the differences in changes in emotional blunting between celecoxib- and placebo-augmented patients, there was a significant difference (*P* = .037) in the change in scores achieved by each of the 2 groups between baseline and week 8 for the ODQ NC subscale, with the celecoxib-augmented group’s estimated mean score change on the NC scale being 2.53 points smaller than the placebo-augmented group’s estimated mean score change over the same period, reflecting that the vortioxetine and placebo–treated group had a greater reduction in reported symptoms of “not caring” (Table 3; Figure 2). This reflects that the placebo-augmented group had a greater reduction in reported apathy than the celecoxib-treated group. No other tested score or subtotal had a significant group difference during this period, indicating that there was a difference in “not caring” but no other ODQ-derived outcomes between vortioxetine plus placebo and vortioxetine plus celecoxib-treated participants by week 8.

Thirdly, when examining the long-term effects of treatment groups on change of emotional blunting between baseline and 3-month and baseline and 6-month follow-up in the individual treatment groups, respectively, there were significant reductions in both treatment groups for both intervals for the ODQ total, RP, NC, and RP-NC scales (Table 4; Figure 2). However, the ODQ GR decreased significantly only in the celecoxib-augmented group and only between baseline and the 3-month follow-up, whereas the ED decreased significantly for both time intervals in the placebo-augmented group only. In addition, the GR-ED decreased significantly for both intervals in the placebo-augmented group but only between baseline and the 3-month follow-up period for the celecoxib-augmented group (Table 4; Figure 2). There were no significant time-by-treatment group interactions when examining change over all 4 time points (Table 4). Finally, there was a significant time-by-treatment interaction for the change between the ODQ ED scale between baseline and 6-month follow-up, favoring the placebo-augmented group. There were no other significant time-by-treatment group interactions between 2 time points for any subscale (data not shown).

Meanwhile, there was no significant change over time in either treatment group for the ODQ AC measure between week 8 and either the 3- or 6-month follow-up appointments (Table 4). However, there was an overall effect of treatment group (F [df] = 68.526 [1,71.953], *P* = .016; Figure 2).

**Figure 3.**
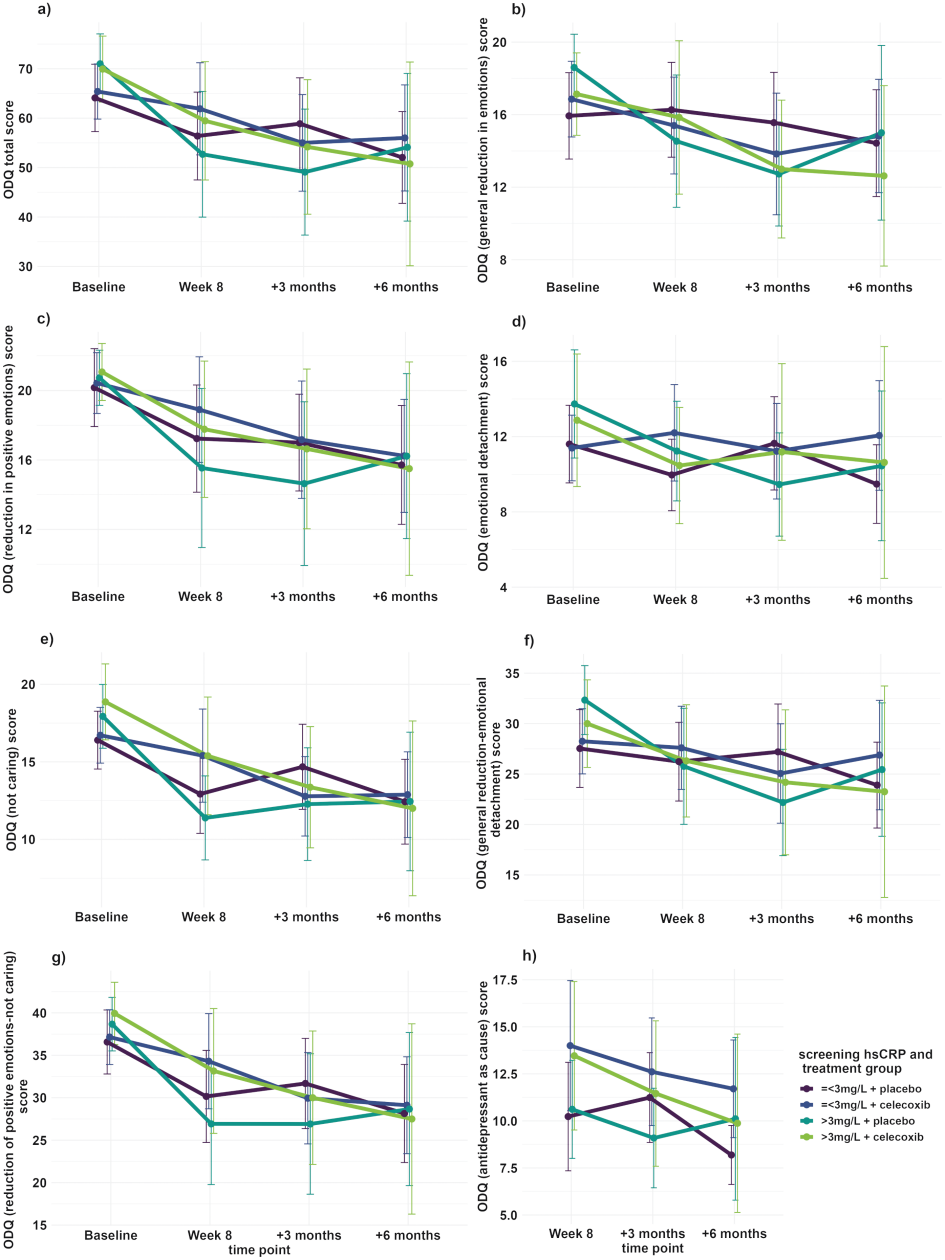
Change over time in Oxford Depression Questionnaire (ODQ) score and subscales over time by treatment group and high sensitivity C-reactive protein (hsCRP) strata. Plots of the mean respective scores at baseline, week 8, 3-month follow-up, and 6-month follow-up time points of participants treated with vortioxetine + placebo and vortioxetine + celecoxib and also stratified by hsCRP ≤3 mg/L and >3 mg/L concentrations at screening for *a* ODQ total score, *b* ODQ general reduction in emotions score, *c* ODQ reduction in positive emotions score, *d* ODQ emotional detachment score, *e* ODQ not caring score, *f* ODQ GR-ED score, *g* ODQ RP-NC score, and *h* ODQ antidepressant as cause score. Error bars = 95% confidence interval (CI).

Finally, assessment of the role of hsCRP concentrations measured at screening showed some associations with changes in ODQ outcomes over time. The significant difference in ODQ ED score between the treatment groups’ change between baseline and 6-month follow-up favoring the placebo-augmented group was replicated in the hsCRP-inclusive model (est [CI] = 3.145 [0.194 to 6.096], *P* = .037; Figure 3D). There were additional significant interaction terms in the models when hsCRP strata was included. For the ODQ total score, there was a statistically significant difference in the magnitude of change between baseline and the 3-month follow-up appointment between participants by hsCRP strata, with participants with screening concentrations >3 mg/L having a greater reduction in ODQ total scores and less reported symptoms of emotional blunting by this time point than participants in the low hsCRP stratum, regardless of treatment group allocation (est [CI] = −14.532 [−26.428 to −2.635], *P* = .017; Figure 3A). Meanwhile, the ODQ GR subscale had statistically significant differences in both the change from baseline to week 8 and the change from baseline to 3-month follow-up according to hsCRP strata, also regardless of treatment group (Figure 3B). Between baseline and week 8, estimated reduction of ODQ GR scores and reported general reduction in emotions of the high hsCRP stratum were 3.942 points lower compared with the high hsCRP stratum (CI = −7.782 to −0.101, *P* = .044), regardless of treatment group. Between baseline and 3-month follow-up, the estimated reduction of ODQ GR scores and reported general reduction in emotions of the high hsCRP stratum were 5.096 points lower compared with the high hsCRP stratum (CI = −9.138 to −1.055, *P* = .014) regardless of treatment group. The high hsCRP stratum also had a significantly larger decrease in ODQ NC score or reported feelings of apathy between baseline and 3-month follow-up than the low hsCRP stratum (est [CI] = −3.824 [−7.419 to −0.228], *P* = .037; Figure 3E). The GR-ED subscale also had a significant difference by hsCRP strata between baseline and 3-month follow-up, with the high hsCRP stratum showing a greater reduction in these symptoms (est [CI] = −8.301 [−14.372 to −2.23], *P* = .008; Figure 3F). There were no statistically significant differences in the change of ODQ RP (Figure 3C), RP-NC (Figure 3G), or AC (Figure 3H) scores from baseline to any later time point according to treatment group or hsCRP strata. There were also no significant overall time-by-hsCRP strata or time-by-treatment group-by-hsCRP strata interactions in any model for any ODQ score or subscale (data not shown), although the overall effect of treatment group on ODQ AC score from the previous model was retained (F [df] = 4.771 [1,70.848], *P* = .032; Figure 3H).

## DISCUSSION

Emotional blunting is an important phenomenon related to both treatment with antidepressants as well as to depression symptomatology, which leads to suffering and potentially compromises adherence to treatment. Our analysis showed a significant decrease in the whole group regarding ODQ total score as well as all subscores from baseline to end of augmentation period of vortioxetine with celecoxib or placebo. This finding supports the hypothesis that antidepressant treatment with vortioxetine improves symptoms of emotional blunting and suggests that emotional blunting should be considered as a symptom of depression. Especially, reduction in positive emotions and not caring, which have been correlated to emotional blunting as a symptom of depression (Price et al., 2012), revealed improvement after 8 weeks of treatment. Previously, vortioxetine has been reported to be efficacious in improving anhedonia in patients with major depressive disorder (McIntyre et al., 2021). It has also been demonstrated to lead to less sexual dysfunction than SSRI treatment (Jacobsen et al., 2015). Given that emotional blunting as a side effect of antidepressants has been related to SSRI-induced sexual dysfunction (Opbroek et al., 2002), favorable effects of vortioxetine on sexual function may also refer to a lessening of treatment-related emotional blunting. In line with this assumption, we have also seen an improvement in subscales (GR-ED), which have been correlated to emotional blunting as a side effect of treatment (Price et al., 2012). The largest trial investigating effects of 10 to 20 mg daily vortioxetine on emotional blunting (n = 143) revealed a strong decrease in ODQ total score (−29.8 points vs −9.4 in the present analysis) (Fagiolini et al., 2021). Although the baseline MADRS score was lower than in the present analysis (25.5 vs 27.5), Fagiolini et al. reported on considerably higher baseline ODQ total score (89.4 vs 66.9), which may explain the greater decrease by the end of the 8-week treatment (Fagiolini et al., 2021) compared with our study. Interestingly, the ODQ total score after 8 weeks was comparable in both studies (59.6 vs 57.5 in the present analysis).

This is the first study, to our knowledge, to report on the impact of augmentation of vortioxetine with an anti-inflammatory agent on emotional blunting in MDD. After stratification into the 2 treatment groups, there was a significant decrease in ODQ total score and in all subscores (except subscore general reduction in emotions, GR) in the vortioxetine plus placebo–treated group, whereas the celecoxib augmented group showed significant improvement only in the ODQ total score and in subscores that represent emotional blunting as a symptom of depression (RP, NC, RP-NC) (Price et al., 2012). Emotional detachment, general reduction in emotions, and GR-ED, which are considered as a side effect of antidepressant medication, did not show significant improvement in the celecoxib augmented group. One explanation for this finding is that celecoxib as a potent CYP2D6 inhibitor (Werner et al., 2003) may have increased plasma levels of vortioxetine, which is a substrate of CYP2D6 (Frederiksen et al., 2022), to higher levels than in the vortioxetine plus placebo group. Positive effects of vortioxetine on emotional blunting may be present only in standard plasma levels with a threshold of plasma level above which higher levels of vortioxetine may attenuate positive treatment effects on emotional blunting in the celecoxib group. Increased levels of vortioxetine may explain unfavorable changes in emotional blunting subscores, which are considered a side effect of antidepressants. An alternative explanation could be that despite known interactions between the 2 medications, the increase in vortioxetine levels may have been less than expected in the celecoxib-augmented group, particularly if members of this group had a fast CYP2D6 metabolizing profile, leading to achieved concentrations below or at the lower end of the therapeutic range. However, the plasma levels were not measured during this study; therefore, these possibilities need to be validated in further investigations. Nevertheless, because side effects of treatment reported in the main outcome paper (Baune et al., 2021) did not differ between the groups, this argues against the hypothesis that celecoxib led to higher or lower vortioxetine levels relative to the placebo-augmented group. Celecoxib itself may have had an unfavorable impact on certain emotional blunting symptoms, explaining the missing improvement in the celecoxib-augmented group in subscores, which are considered as a side effect of antidepressant medication. Although nonsteroidal anti-inflammatory drugs are known to cause psychiatric symptoms in anti-inflammatory treatment (Onder et al., 2004), there is no previous published study on the relationship between celecoxib or other nonsteroidal anti-inflammatory drugs and emotional blunting. There is also no previous research investigating whether low-grade inflammation plays a role in the pathogenesis of emotional blunting in depressed individuals. Hence, further studies are needed to investigate whether anti-inflammatory treatment of depression with celecoxib is associated with emotional blunting as a side effect and whether emotional blunting is related to inflammation.

The analysis did not show any time-by-treatment group effect after 8 weeks in ODQ total score and subscores other than NC (“not caring”), which revealed greater improvement of self-reported apathy in the placebo-augmented group than in the celecoxib group. Given these findings, which need confirmation in independent and larger cohorts, our study does not support the use of celecoxib as an augmentation to vortioxetine in patients where emotional blunting is a prominent clinical feature. However, our findings need replication because there is no other report on the effect of celecoxib or other anti-inflammatory agent on emotional blunting in the literature.

Meanwhile, hsCRP concentrations greater than 3 mg/L at screening were associated with larger score decreases and greater clinical recovery in multiple symptom areas between baseline and later time points compared with those with 3 mg/L or less hsCRP at screening, regardless of treatment group allocation. As patients who have evidence of inflammation associated with their MDD have been observed to be more likely to be resistant to treatment (Strawbridge et al. 2015) and to have a more severe course of illness (Köhler-Forsberg et al. 2017), it is unexpected that these participants would appear to have better outcomes than participants without evidence of inflammation associated with their MDD. Although this may be suggestive of utility of either vortioxetine or celecoxib treatment on emotional blunting symptoms in MDD, it is possible that these associations were present due to the elevated hsCRP stratum’s slightly higher scores recorded at baseline in both treatment groups compared with the hsCRP ≤3 mg/L stratum for 7 out of the 8 ODQ-derived measures. Although this would still suggest that patients with more severe symptomatology could benefit, there may also be a threshold effect below which scores are unlikely to decrease. As seen in Figure 3, the mean ODQ RP, NC, and RP-NC scores for all 4 groups are closely clustered at 6-month follow-up visit.

The present study is also the only study, to our knowledge, that reports on long-term treatment effects on emotional blunting after 8 total months of antidepressant treatment. Here we have seen significant improvements in both treatment groups for the 3- and 6-month follow-up study periods in the ODQ total score as well as in subscores that are considered to represent emotional blunting as a symptom of depression (RP, NC, RP-NC), whereas changes in subscores related to emotional blunting as a side effect of treatment (GR, ED, GR-ED) revealed less clear long-term changes. These findings support the hypothesis that vortioxetine leads to improvement in emotional blunting total score in both long- and short-term use as an antidepressant. Our results also support the assumption of the validation study (Price et al., 2012) that general reduction in emotions and emotional detachment may represent a side effect of treatment because we have seen less improvement in these domains during treatment course. Further effort must be taken in the development of antidepressant strategies to improve these domains of emotional blunting.

There was no significant change in the antidepressant as cause (AC) subscore, which was assessed at 8 weeks, 3-month follow-up, and 6-month follow-up. However, AC scores were significantly higher in the vortioxetine plus celecoxib–treated group than in the vortioxetine plus placebo group, supporting the hypothesis of increased negative vortioxetine effects due to pharmacokinetic interaction between vortioxetine and celecoxib, as discussed before.

Strengths of the present study are the randomized, controlled design and the use of the ODQ including all subscores, which is a validated tool to assess emotional blunting in depressed patients. The report on long-term impact of antidepressant treatment after 3 and 6 months as well as the investigation of the effect of anti-inflammatory treatment on emotional blunting are unique contributions to the existing literature. The main limitation of the study is that there is no group that received placebo without vortioxetine. Thus, it is not possible to differentiate between a treatment effect or side effects of vortioxetine and effect of treatment expectations of study participants on the outcome measures. Furthermore, a plurality of measures tested, including those already published, increases the risk of type I errors, so this secondary analysis must be considered exploratory. The post-RCT-phase measurement was 2 weeks after cessation of celecoxib/placebo, which, depending on the causal mechanisms, may have reduced the difference between the 2 groups compared with if a measurement had been taken at week 6. The present analysis does not report on the impact of pretreatment stratification of participants in the hsCRP strata depression with and without low-grade inflammation, as described in the study protocol (Fourrier et al., 2018). Finally, plasma concentrations of vortioxetine during the study have not been measured, and therefore causal explanations for relationships between emotional blunting, vortioxetine, and celecoxib cannot yet be confirmed.

In summary, this analysis confirmed beneficial effects of vortioxetine treatment on emotional blunting, which may have been weakened by celecoxib augmentation due to pharmacokinetic enzyme interactions.

## Data Availability

The data underlying this article cannot be shared publicly to protect the privacy of individuals that participated in the study. The data will be shared on reasonable request to the corresponding author.

## Notes

This work was supported by the Fay Fuller Foundation.

## Interest Statement

H. Lundbeck A/S did not exert any influence on the study conception, study design, data collection, data analyses, or manuscript preparation and publication. The funding agency, the Fay Fuller Foundation, did not exert any influence on the study conception, study design, data collection, data analyses and manuscript preparation, or publication. Dr Erhan Kavakbasi received speaker and advisor honoraria from LivaNova, Janssen-Cilag, and Aristo Pharma. A/Professor Hikaru Hori received speaker fees from Sumitomo Pharma, Otsuka, Meiji-Seika Pharma, MSD K.K., Pfizer, Janssen Pharmaceutical, Shionogi, and Takeda Pharmaceutical. A/Professor K. Oliver Schubert received speaker/consultation fees from Janssen-Cilag and received research funding from Lundbeck, Janssen-Cilag, and Gilead. Professor Bernhard T. Baune received speaker/consultation fees from AstraZeneca, Lundbeck, Pfizer, Takeda, Servier, Bristol Myers Squibb, Otsuka, LivaNova, Biogen, Angelini, and Janssen-Cilag. Ms Emma Sampson, Dr Natalie Mills, and Dr Célia Fourrier report no financial relationships with commercial interests.
